# Correction to “Genotype‐Specific Safety and Pharmacokinetics of Cannabidiol in Healthy Volunteers”

**DOI:** 10.1111/cts.70506

**Published:** 2026-02-13

J. Etkins, G. C. So, J. B. L. Lu, et al., “Genotype–Specific Safety and Pharmacokinetics of Cannabidiol in Healthy Volunteers,” *Clinical and Translational Science* 19 (2026): e70455, https://doi.org/10.1111/cts.70455.

In the article cited above, Figures 4 (single dose) and 5 (steady state) were transposed. Figure 5 was published in the place of Figure 4 and vice versa.

The correct labeling is as follows:

**Figure 4**

**FIGURE 4:**
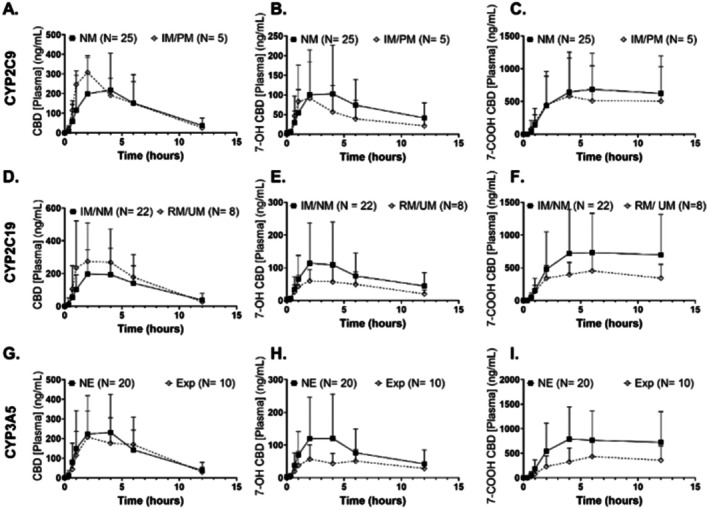
Mean (and standard deviation) plasma concentration versus time graphs are depicted for (A, D, G) CBD, (B, E, H) 7‐OH CBD, and (C, F, I) 7‐COOH CBD after a single dose of CBD was administered. Groups are compared according to metabolic enzyme genotype for (A–C) CYP2C9, (D–F) CYP2C19, and (G–I) CYP3A5. Significance was tested by Student's *t‐*test.

**Figure 5**

**FIGURE 5:**
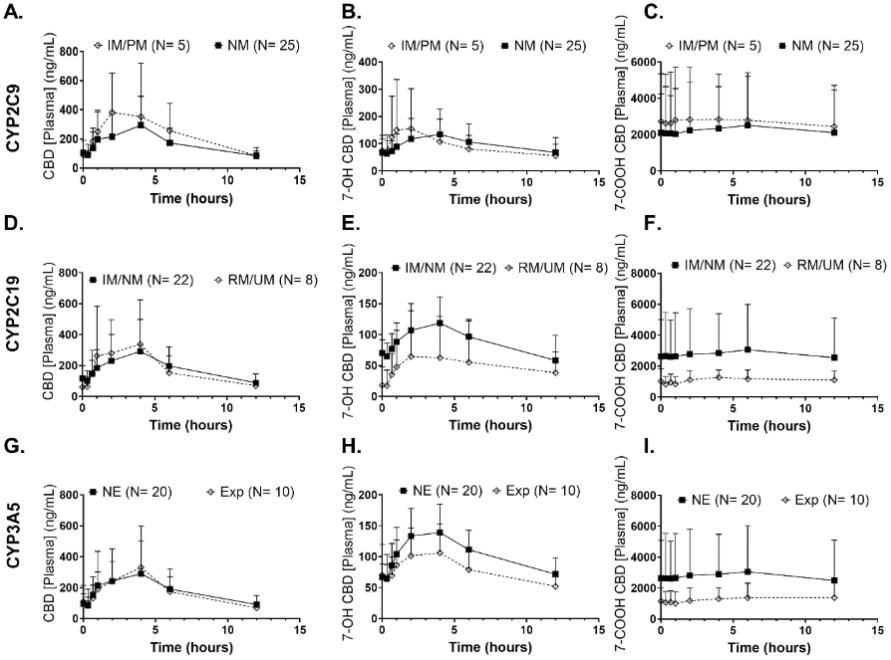
Mean (and standard deviation) plasma concentration versus time graphs are depicted for (A, D, G) CBD, (B, E, H) 7‐OH CBD, and (C, F, I) 7‐COOH CBD at steady state after 11 days of CBD 5 mg/kg twice daily was taken. Groups are compared according to metabolic enzyme genotype for (A–C) CYP2C9, (D–F) CYP2C19, and (G–I) CYP3A5. Significance was tested by Student's *t‐*test.

